# Supplementary material for: Flexibility in choosing decision policies in gathering discrete evidence over time
Source: PLoS One. 2025 Jan 14;20(1):e0316320. doi: 10.1371/journal.pone.0316320 (PMC11731777; doi:10.1371/journal.pone.0316320)
Supplement: Attachment — Submitted filename: PlosOne_Flexibility.docx [file pone.0316320.s001.docx]

The study “Flexibility in Choosing Decision Policies in Gathering Discrete Evidence Over Time“ offers insights into the brain’s adaptive strategies in decision-making across different sensory modalities. It highlights the brain's ability to maintain a consistent method of integrating evidence while flexibly adjusting how that evidence is weighted based on the context provided by the sensory modality. This underscores the brain's flexibility in optimizing decision-making processes in diverse environments.

By using the same object categorization task (faces vs. cars) and assuming linear integration as the decision solution, the authors attribute any differences in performance to changes in decision policies.

The primary discovery of this study is that while the brain utilizes a consistent decision solution (linear integration of evidence) across different sensory modalities (visual and auditory), it flexibly adapts its decision policies based on the sensory context. Specifically:

- Visual Tasks: The brain places greater weight on the first pulse of evidence, making the initial stimulus more influential in decision-making.
- Auditory Tasks: The brain treats both pulses of evidence equally, showing no preference for the sequence in which auditory information is received.

The authors conclude that the brain employs a consistent method (decision solution) of integrating evidence over time across different sensory modalities but adapts its decision policies (strategies for weighting evidence) based on the context provided by the sensory modality. This flexibility allows the brain to optimize decision-making processes in dynamic environments and suggests that cognitive strategies are not fixed but can be tailored to the demands of the situation.

Overall, the manuscript presents a meaningful contribution to the field of cognitive neuroscience and perceptual decision-making by demonstrating the brain’s flexibility in decision policies across sensory modalities. While the core findings are compelling, addressing the limitations mentioned below would strengthen the manuscript.

**Main comments:**

1. Provide clearer definitions of key terms, particularly decision policies and decision solutions, early in the manuscript.

2. Elaborate on the implications of the findings and potential neural mechanisms involved. e.g.,

What is the interpretation of a lack of effect for inter-pulse interval?

or,

Why in visual, but not auditory, does the sequence of pulse strength matter?

Also please justify temporal gap selection: why were sub-second intervals chosen, and not larger inter pulse intervals?

3. Sample size (15 subjects with 2 being authors) is relatively small. Please consider adding more subjects, or acknowledge the sample size limitations and potential biases, suggesting ways future research can address these issues.

4. To make it easier for the reader to skim the figures and understand the paper, please change all figure titles to complete sentences that convey the main takeaway of the figure; e.g. Figure 3 title should be like:

"Participants' sensory discrimination improves with increased combined stimulus strength in both visual and auditory tasks"

**Minor comments:**

1. Remove the extra "and" in figure 3 title. (made bold below):

Figure 3. Participants' sensitivity (d-prime) in single and double-pulse trials **and** in both visual and auditory tasks

2. Figure 4 title: remove the dash after decision
