# Supplementary material for: Flexibility in choosing decision policies in gathering discrete evidence over time
Source: PLoS One. 2025 Jan 14;20(1):e0316320. doi: 10.1371/journal.pone.0316320 (PMC11731777; doi:10.1371/journal.pone.0316320)
Supplement: Attachment — Submitted filename: Response to Reviewers.docx [file pone.0316320.s002.docx]

Dear Reviewer,

Thank you sincerely for your thoughtful and thorough review of our manuscript. We greatly appreciate the time and effort you dedicated to reading our work and providing insightful comments and suggestions. Your feedback has been invaluable in helping us enhance the quality and clarity of the manuscript.

We have carefully addressed each of your comments, both specific and general. Below, you will find a detailed, point-by-point response to each comment. We hope these revisions meet your expectations and contribute to a more robust manuscript.

Warm regards,

Reza Ebrahimpour

**Review Comments to the Author**

**Reviewer #1: The study “Flexibility in Choosing Decision Policies in Gathering Discrete Evidence Over Time“ offers insights into the brain’s adaptive strategies in decision-making across different sensory modalities. It highlights the brain's ability to maintain a consistent method of integrating evidence while flexibly adjusting how that evidence is weighted based on the context provided by the sensory modality. This underscores the brain's flexibility in optimizing decision-making processes in diverse environments.

By using the same object categorization task (faces vs. cars) and assuming linear integration as the decision solution, the authors attribute any differences in performance to changes in decision policies.

The primary discovery of this study is that while the brain utilizes a consistent decision solution (linear integration of evidence) across different sensory modalities (visual and auditory), it flexibly adapts its decision policies based on the sensory context. Specifically:**

**- Visual Tasks: The brain places greater weight on the first pulse of evidence, making the initial stimulus more influential in decision-making.**

**- Auditory Tasks: The brain treats both pulses of evidence equally, showing no preference for the sequence in which auditory information is received.**

**The authors conclude that the brain employs a consistent method (decision solution) of integrating evidence over time across different sensory modalities but adapts its decision policies (strategies for weighting evidence) based on the context provided by the sensory modality. This flexibility allows the brain to optimize decision-making processes in dynamic environments and suggests that cognitive strategies are not fixed but can be tailored to the demands of the situation.**

**Overall, the manuscript presents a meaningful contribution to the field of cognitive neuroscience and perceptual decision-making by demonstrating the brain’s flexibility in decision policies across sensory modalities. While the core findings are compelling, addressing the limitations mentioned below would strengthen the manuscript.**

**Main comments:**

**1: Provide clearer definitions of key terms, particularly decision policies and decision solutions, early in the manuscript.**

**Authors:** Thank you for your suggestion to clarify key terms. Below, we present more precise definitions of "decision policies" and "decision solutions," supported by a broader range of examples with references.

Flexibility in decision-making can be categorized into three levels: flexibility in task solutions, flexibility in decision policies, and flexibility in stimulus-action mapping (Okazawa & Kiani, 2023). At the highest level, decision-makers can adopt different solutions for a task, such as integrating, differentiating, or even disregarding sensory inputs depending on the context. For example, evidence accumulation is the optimal solution for tasks involving the discrimination or categorization of stable sensory information (Waskom, & Kiani, 2018; Stine et al., 2013). However, other task conditions may call for different approaches. When comparing the magnitudes of two stimuli, subtracting inputs is more effective than integrating them (Romo & Lafuente, 2013; Machens et al., 2005), whereas, for detection tasks against a stable background, differentiation is a better strategy than integration. At the next level, decision-makers adjust decision policies by modifying parameters within decision-making models to achieve optimal outcomes. These adjustments support flexible computation, enabling decision-makers to weigh sensory inputs, prioritize evidence, assess costs and rewards (Doi et al., 2020), and manage urgency to respond (Forstmann et al., 2008). Finally, flexibility in stimulus-action mapping allows decision-makers to associate sensory inputs with appropriate actions, emphasizing the adaptable nature of decision-making processes.

These definitions have been incorporated into the relevant paragraph in the introduction. Below is the revised version of that paragraph.

**“****In the literature, flexibility in decision-making, often referred to as context dependency, reflects the brain's ability to adjust its behavior and neural activity across different tasks to meet varying demands. This adaptability is organized into three levels, each representing distinct types of flexibility that enhance decision making performance (Okazawa & Kiani, 2023).** **At the highest level, decision-makers can adopt different solutions for a task, such as integrating, differentiating, or even disregarding sensory inputs depending on the context. For example, evidence accumulation is the optimal solution for tasks involving the discrimination or categorization of stable sensory information (Waskom, & Kiani, 2018; Stine et al., 2013). However, other task conditions may call for different approaches. When comparing the magnitudes of two stimuli, subtracting inputs is more effective than integrating them (Romo & Lafuente, 2013; Machens et al., 2005), whereas, for detection tasks against a stable background, differentiation is a better strategy than integration. At the next level, decision-makers adjust decision policies by modifying parameters within decision-making models to achieve optimal outcomes. These adjustments support flexible computation, enabling decision-makers to weigh sensory inputs, prioritize evidence, assess costs and rewards (Doi et al., 2020), and manage urgency to respond (Forstmann et al., 2008). Finally, perceptual decisions involve mapping sensory stimuli to actions, but existing decision-making models typically assume a fixed stimulus-action association and focus on evidence integration rather than flexible stimulus-action mapping.** **Together, these levels of flexibility demonstrate that decision-making is not simply about processing information but involves a multi-layered, adaptive system capable of dynamically adjusting solutions and policies in response to the immediate context, thereby enabling more effective navigation of complex and real situations.”**

- Okazawa, G., & Kiani, R. (2023). Neural mechanisms that make perceptual decisions flexible. Annual review of physiology, 85(1), 191-215.
- Waskom, M. L., & Kiani, R. (2018). Decision making through integration of sensory evidence at prolonged timescales. Current Biology, 28(23), 3850-3856
- Stine, G. M., Zylberberg, A., Ditterich, J., & Shadlen, M. N. (2020). Differentiating between integration and non-integration strategies in perceptual decision making. *Elife*, *9*, e55365.
- Romo, R., & de Lafuente, V. (2013). Conversion of sensory signals into perceptual decisions. Progress in neurobiology, 103, 41-75.
- Machens, C. K., Romo, R., & Brody, C. D. (2005). Flexible control of mutual inhibition: a neural model of two-interval discrimination. *Science*, *307*(5712), 1121-1124.
- Doi, T., Fan, Y., Gold, J. I., & Ding, L. (2020). The caudate nucleus contributes causally to decisions that balance reward and uncertain visual information. Elife, 9, e56694.
- Forstmann, B. U., Dutilh, G., Brown, S., Neumann, J., Von Cramon, D. Y., Ridderinkhof, K. R., & Wagenmakers, E. J. (2008). Striatum and pre-SMA facilitate decision-making under time pressure. Proceedings of the National Academy of Sciences, 105(45), 17538-17542.

**2- Elaborate on the implications of the findings and potential neural mechanisms involved. e.g., What is the interpretation of a lack of effect for inter-pulse interval?**

**or, Why in visual, but not auditory, does the sequence of pulse strength matter?**

**Also please justify temporal gap selection: why were sub-second intervals chosen, and not larger inter pulse intervals?**

**Authors:** We appreciate the reviewer's insightful comments and the opportunity to elaborate on our findings and their implications. Below, we address each of the points raised, including additional relevant literature to strengthen our explanations.

**a) What is the interpretation of a** **lack of effect for inter-pulse interval?**

**Authors:** The lack of an effect of inter-pulse interval on decision accuracy in both visual and auditory tasks suggests that the brain is capable of maintaining and integrating sensory evidence over temporal gaps of up to approximately one second without significant decay. This finding underscores the robustness of the neural mechanisms responsible for preserving sensory information from the initial pulse across sub-second intervals. Such robustness is crucial for effective decision-making in a dynamic environment and enables the brain to adapt to the irregular timing of sensory signals typically encountered in everyday life. Moreover, this finding aligns with previous research demonstrating that humans can integrate sensory evidence over temporal gaps without substantial loss of information (Kiani et al., 2013; Waskom & Kiani, 2018; Tohidi-Moghaddam et al., 2019).

To explain the observed lack of effect for the inter-pulse interval, we have added the green text in the following paragraph of the manuscript’s discussion section.

**“Crucially in this work, we were able to study flexibility in choosing decision policies. In all decisions, decision-makers must determine the context, set up the solution, decision policies, and associate sensory inputs to actions. In this work, it was shown that linear integration is the appropriate solution for both visual and auditory tasks. Additionally, both tasks have the same action. Therefore, it would be possible to study flexibility in decision policy. Initially, we observed the same policy in both visual and auditory tasks. The accuracy of double-pulse trials was independent of the temporal gap between the two stimuli. This finding aligns with the effect of the inter-pulse interval on the double-pulse motion direction discrimination task in prior research by Kiani et al., 2013.** **This result shows that the brain can retain and integrate sensory information effectively over short temporal gaps (up to one second) without loss of accuracy, highlighting robust neural mechanisms for maintaining sensory evidence. This ability also supports adaptive decision-making when sensory information is temporarily absent** **for varying short durations. Moreover, although previous research has shown that the sequence of visual motion pulses can affect choice accuracy, we cannot extend this phenomenon to the auditory object categorization task. In the auditory task, both pulses hold equal importance, while in the visual mode, the first pulse is more important than the second, which is in contrast to the prior result with random dot motion stimulus. Thus, the human brain can flexibly change its decision policies when facing different contexts, which helps it achieve optimal performance in any task.”**

**Potential Neural Mechanisms:**

The maintenance of sensory evidence may invol ve persistent activity in neural circuits, particularly within prefrontal or parietal cortices. Leon and Shadlen (2003) demonstrated that neurons in the posterior parietal cortex can sustain representations of sensory information over delays, facilitating integration processes. Such sustained neural activity could keep the representation of the first pulse active during the inter-pulse interval, allowing it to be effectively integrated with the second pulse upon its arrival. Moreover, the integration over temporal gaps likely engages working memory mechanisms. The dorsolateral prefrontal cortex and posterior parietal cortex are known to support the temporary storage and manipulation of sensory information, which could underlie the observed integration across intervals (Gold & Shadlen, 2007; Curtis & D'Esposito, 2003). Recent research by Azizi and Ebrahimpour (2023) provides valuable insights into the neural mechanisms involved in integrating sensory evidence presented with temporal gaps. They demonstrated that decisions based on the accumulation of evidence from separated cues over time are best explained by the interplay of recurrent cortical dynamics of centro-parietal and frontal brain areas. This interplay enables the brain to maintain and combine sensory information over delays efficiently.

Based on the above points, we have added the following paragraph to the discussion section and inserted the necessary references:

**“****Previous studies shed light on the neural mechanisms that enable the brain to efficiently integrate sensory information across temporal gaps. It was shown that persistent neural activity in regions such as the posterior parietal cortex allows sensory representations to be maintained during delays, supporting the integration process (Leon & Shadlen, 2003). Additionally, working memory processes, particularly in the dorsolateral prefrontal and posterior parietal cortices, facilitate temporary storage of information for effective combination across intervals (Gold & Shadlen, 2007; Curtis & D’Esposito, 2003. Expanding on these mechanisms, Azizi and Ebrahimpour (2023)** **propose a model that emphasizes the interplay of recurrent cortical dynamics within the centro-parietal and frontal brain areas. This dynamic interaction facilitates the efficient maintenance and integration of sensory evidence over time, ensuring accurate and cohesive decision-making.”**

- Kiani, R., Churchland, A. K., & Shadlen, M. N. (2013). Integration of direction cues is invariant to the temporal gap between them. Journal of Neuroscience, 33(42), 16483-16489.
- Waskom, M. L., & Kiani, R. (2018). Decision making through integration of sensory evidence at prolonged timescales. Current Biology, 28(23), 3850-3856.
- Tohidi-Moghaddam, M., Zabbah, S., Olianezhad, F., & Ebrahimpour, R. (2019). Sequence-dependent sensitivity explains the accuracy of decisions when cues are separated with a gap. Attention, Perception, & Psychophysics, 81, 2745-2754.
- Leon, M. I., & Shadlen, M. N. (2003). Representation of time by neurons in the posterior parietal cortex of the macaque. Neuron, 38(2), 317-327.
- Gold, J. I., & Shadlen, M. N. (2007). The neural basis of decision making. Annu. Rev. Neurosci., 30(1), 535-574.
- Curtis, C. E., & D'Esposito, M. (2003). Persistent activity in the prefrontal cortex during working memory. Trends in cognitive sciences, 7(9), 415-423.
- Azizi, Z., & Ebrahimpour, R. (2023). Explaining Integration of Evidence Separated by Temporal Gaps with Frontoparietal Circuit Models. Neuroscience, 509, 74-95.

**b) Why in visual, but not auditory, does the sequence of pulse strength matter?**

**Authors:** The differential effect of pulse sequence in the visual and auditory tasks likely reflects modality-specific processing characteristics in the brain. Our finding that pulse sequence impacts visual but not auditory processing can be explained by modality-specific neural pathways and the mechanisms of temporal integration. The visual system is known for its sensitivity to sequential patterns and is adept at processing time-dependent information in a structured way. This sensitivity is associated with cortical regions such as the visual cortex, where temporal and spatial dynamics are more finely tuned to detect changes and sequences. Auditory pathways, in contrast, often employ a rapid, more continuous processing mode optimized for different types of temporal coding (e.g., integration over rapid sound sequences without much variation)(Okazawa and Kiani, 2023).

**Visual Task:**

In the visual modality, we observed that the first pulse had a greater influence on the decision than the second pulse. This may be due to visual stimuli often having enduring representations and may also capture attention more effectively, leading to enhanced encoding and greater influence on the decision-making process (Carrasco, 2011). Subsequent stimuli might be subject to reduced attentional resources or might be interpreted in the context of the initial percept. Finally, sustained activity in visual cortical areas could preferentially maintain the information from the first pulse. The ventral visual stream, involved in object recognition, may prioritize initial inputs, affecting the integration process (Di Lollo et al., 2000).

**Auditory Task:**

In contrast, the auditory system appears to weigh both pulses equally. Auditory information is inherently temporal and transient, and the auditory system specializes in processing rapid sequences of sounds (Recanzone, 2009). The auditory system has also a higher temporal resolution than the visual system, allowing for finer discrimination of temporal sequences. This might result in both pulses being integrated with similar weights. Moreover, auditory cortical areas may process sequential sounds in a manner that facilitates equal integration of inputs. Unlike visual memory, auditory memory may not exhibit the same primacy effects observed in visual memory. Cowan et al. (1998) suggested that auditory working memory might not show proactive interference to the same extent, leading to equal weighting of sequential inputs.

Based on the points mentioned, we have added the following paragraph to the discussion section:

**“The differential effect of pulse sequencing observed between visual and auditory tasks can be attributed to modality-specific processing pathways. In visual tasks,** **visual stimuli often have enduring representations, and the first pulse might influence subsequent processing. Moreover, the initial visual stimulus may capture attention more strongly, leading to better encoding and a greater impact on decision-making, while later stimuli may receive less attention or be interpreted relative to the first (Carrasco, 2011). Conversely, auditory processing does not show this primacy effect, likely due to its high temporal resolution and capability for continuous integration, which facilitates equal weighting of sequential inputs (Recanzone, 2009; Cowan et al., 1998).”**

- Okazawa, G., & Kiani, R. (2023). Neural mechanisms that make perceptual decisions flexible. Annual review of physiology, 85(1), 191-215.
- Di Lollo, V., Enns, J. T., & Rensink, R. A. (2000). Competition for consciousness among visual events: the psychophysics of reentrant visual processes. *Journal of Experimental Psychology: General*, *129*(4), 481.
- Carrasco, M. (2011). Visual attention: The past 25 years. *Vision research*, *51*(13), 1484-1525.
- Recanzone, G. H. (2009). Interactions of auditory and visual stimuli in space and time. Hearing research, 258(1-2), 89-99.
- Cowan, N. (1998). Visual and auditory working memory capacity. *Trends in cognitive sciences*, *2*(3), 77.

**c) Also, please justify** **temporal gap selection: why were sub-second intervals chosen, and not larger inter-pulse intervals?**

**Authors:** We outline our reasons for selecting sub-second temporal gaps in the points below.

**Consistency with Previous Research:** We selected inter-pulse intervals ranging from 0 to 1080 ms to match those used in Kiani et al. (2013), titled "Integration of Direction Cues Is Invariant to the Temporal Gap between Them." Our experimental paradigm closely mirrors this study, differing only in the use of natural stimuli for our visual and auditory tasks, compared to the random dot motion stimuli used in the original study. This alignment allows for direct comparisons between these three conditions.

**Relevance to Perceptual Decision-Making:** Sub-second intervals are ecologically valid and represent the timescales on which perceptual decisions are typically made. In natural environments, sensory information often arrives in rapid succession, requiring decisions based on closely spaced inputs. However, a study by Waskom & Kiani (2018), titled “Decision Making through Integration of Sensory Evidence at Prolonged Timescales”, examined longer temporal gap durations between 2-8 seconds and found that longer gaps did not reduce accuracy or cause information leakage. Future research could enhance our findings by studying prolonged gap durations across different sensory modalities, as mentioned in the Limitations and Future Work section of our manuscript.

**Avoidance of Additional Cognitive Processes:** Larger inter-pulse intervals could engage different cognitive mechanisms, such as long-term memory retrieval or attentional shifts, which are beyond the scope of our study. Previous research indicated that the temporal integration window for perceptual decision-making spans several hundred milliseconds (Gold & Shadlen, 2007). By keeping intervals within the sub-second range, we focused on short-term sensory integration without confounding factors.

Finally, to clarify temporal gap selection, we added the green part in the following paragraph of the Results section of our manuscript.

**“In our study, we utilized a brief stimulus presentation of 50 ms in each pulse, shorter than the 120 ms presentation used in the double pulse motion direction discrimination task conducted by Kiani et al (2013). However, it is important to note that the gap duration between pulses varied between 0 ms and 1080 ms, similar to the experimental design employed by Kiani et al.** **By selecting sub-second inter-pulse intervals, we ensured our study remained focused on temporal dynamics relevant to perceptual decision-making processes. This choice allowed us to investigate how the brain integrates discrete sensory evidence over short timescales, essential for understanding perceptual decision-making in many real-world conditions. The previous findings demonstrated that performance could be modelled as a linear integration of sensory evidence (more precisely, logistic regression), independent of the interval between cues. Building upon this solution and decision policy, we explored how varying gap durations between pulses impact the decision-making process in visual and auditory object categorization tasks.”**

- Gold, J. I., & Shadlen, M. N. (2007). The neural basis of decision making. Annu. Rev. Neurosci., 30(1), 535-574.
- Kiani, R., Churchland, A. K., & Shadlen, M. N. (2013). Integration of direction cues is invariant to the temporal gap between them. Journal of Neuroscience, 33(42), 16483-16489.
- Waskom, M. L., & Kiani, R. (2018). Decision making through integration of sensory evidence at prolonged timescales. Current Biology, 28(23), 3850-3856.

3- **Sample size (15 subjects with 2 being authors) is relatively small. Please consider adding more subjects, or acknowledge the sample size limitations and potential biases,** **suggesting ways future research can address these issues.**

**Authors:** We appreciate your comments regarding the sample size. We acknowledge that the sample size of 15 participants, with 2 being authors, is relatively small. However, this approach aligns with many perceptual decision-making studies which often rely on a limited number of subjects while gathering extensive trial data. Given the perceptual nature of the task, it is expected that the principles of perceptual decision-making will remain consistent across all subjects. In our study, we collected 672 trials from each participant in each modality. Below, we provide a table summarizing the sample sizes from similar studies:

| **Article** | **Authors** | **Sample size** | **Number of trials** | **Year** | **Journal** |
| --- | --- | --- | --- | --- | --- |
| Elapsed decision time affects the weighting of prior probability in a perceptual decision task | Hanks, et al. | 2 human subjects | ≥ 2400 per subject | 2011 | Journal of Neuroscience |
| Confidence is the bridge between multi-stage decisions | Van Den Berg, et al. | 3 human subjects | 5544  per subject | 2016 | Current Biology |
| Buildup of Choice-Predictive Activity in Human Motor Cortex during Perceptual Decision Making | Donner, et al. | 4 human subjects | ≥ 3200 per subject | 2009 | Current Biology |
| Analysis of sequential effects on choice reaction times | Remington | 5 human subjects | 1000  per subject | 1969 | Journal of experimental psychology |
| A common mechanism underlies changes of mind about decisions and confidence | Van Den Berg,, et al. | 6 human subjects | 5000-9023  per subject | 2016 | Elife |
| Choice Certainty Is Informed by Both Evidence and Decision Time | Kiani, et al. | 6 human subjects | 1400-300 per subject | 2014 | Neuron |

Our findings also align with those of previous studies (e.g., Kiani et al., 2013; Tohidi-Moghaddam et al., 2019), lending support to the validity of our results.

To address concerns regarding sample size, we have added a paragraph titled 'Limitations and Future Work' following the conclusion. This paragraph outlines potential biases and suggests ways for future research to address these issues. We have omitted the discussion of future work from the conclusion paragraph and incorporated it into this new paragraph instead.

**“****Limitations and Future Work**

**To enhance statistical power and generalizability, future studies should include more participants and avoid author involvement to reduce potential bias. Furthermore, it would be valuable to investigate the impact of other sensory modalities, such as olfactory and haptic senses, on the perceptual decision-making process. This exploration could provide further insights into how different sensory modalities influence the brain's flexibility in selecting decision policies in multisensory environments.** **Longer temporal gap durations could also be considered to encompass a broader range of conditions in future research.”**

- Kiani, R., Churchland, A. K., & Shadlen, M. N. (2013). Integration of direction cues is invariant to the temporal gap between them. Journal of Neuroscience, 33(42), 16483-16489.
- Tohidi-Moghaddam, M., Zabbah, S., Olianezhad, F., & Ebrahimpour, R. (2019). Sequence-dependent sensitivity explains the accuracy of decisions when cues are separated with a gap. *Attention, Perception, & Psychophysics*, *81*, 2745-2754.

4- **To make it easier for the reader to skim the figures and understand the paper, please change all figure titles to complete sentences that convey the main takeaway of the figure; e.g. Figure 3 title should be like:**

**"Participants' sensory discrimination improves with increased combined stimulus strength in both visual and auditory tasks"**

**Authors:** Thank you for your insightful suggestion to improve the manuscript's readability and clarity. To help readers quickly understand the key findings, we have revised all figure titles into complete sentences. The updated titles are provided below:

**Figure 1**

**Revised Title:** Participants categorize noisy images or sounds in a double-pulse object categorization task presented in visual and auditory modalities.

**Figure 2**

**Revised Title:** Accuracy increases with stimulus strength in both single and double-pulse trials for visual and auditory tasks.

**Figure 3**

**Revised Title:** Participants' sensory discrimination improves with increased combined stimulus strength in both visual and auditory tasks.

**Figure 4**

**Revised Title:** A shared decision policy is applied to the temporal gap, while distinct policies are applied to the sequence of pulses in visual and auditory tasks.

**Figure 5**

**Revised Title:** Participants' confidence increases with aggregated stimulus strength and correlates with accuracy in both visual and auditory tasks.

**Minor comments:**

**1. Remove the extra "and" in figure 3 title. (made bold below):**

**Figure 3. Participants' sensitivity (d-prime) in single and double-pulse trials and in both visual and auditory tasks**

**2. Figure 4 title: remove the dash after decision.**

**Authors:** We appreciate your careful feedback on refining the figure titles. Following your previous suggestion, we modified the titles into full sentences for clarity, while considering your additional minor comments.
